# Au naturale: use of biologically derived cyclic di-nucleotides for cancer immunotherapy

**DOI:** 10.1098/rsob.210277

**Published:** 2021-12-15

**Authors:** Christopher M. Waters

**Affiliations:** Department of Microbiology and Molecular Genetics, Michigan State University, 5180 Biomedical and Physical Sciences, 567 Wilson Road, East Lansing, MI 48824, USA

**Keywords:** STING, cancer, cyclic di-nucleotides, immunotherapy

## Abstract

Cyclic di-nucleotides (CDNs) are widespread second messenger signalling molecules that regulate fundamental biological processes across the tree of life. These molecules are also potent modulators of the immune system, inducing a Type I interferon response upon binding to the eukaryotic receptor STING. Such a response in tumours induces potent immune anti-cancer responses and thus CDNs are being developed as a novel cancer immunotherapy. In this review, I will highlight the use, challenges and advantages of using naturally occurring CDNs to treat cancer.

## Graphical abstract

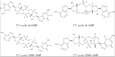

## 1. Introduction

A rationale for pursuing basic science research, or the pursuit of knowledge for its own sake, is that it is unpredictable which fundamental discoveries will lead to the development of useful clinical innovations. Cyclic di-nucleotide (CDN) molecules are a classic example that supports this justification. These signalling molecules, which are found across the tree of life, were initially described as global regulators of microbial physiology, but we now understand they are also potent modulators of the immune system. Such activity has exciting potential to manipulate the immune system to treat disease such as cancer.

**Figure 1.**
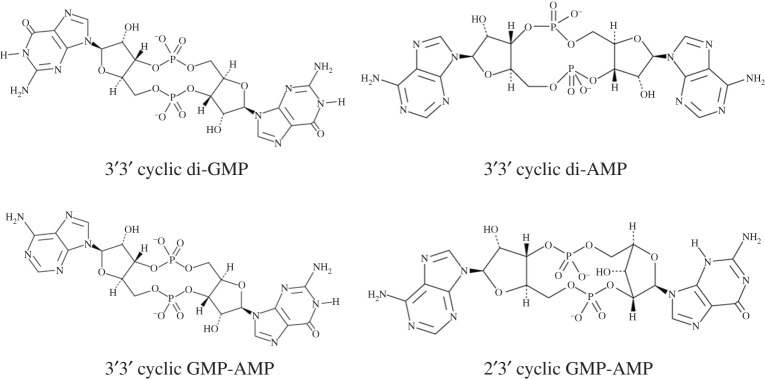
The chemical structure of the four major CDNs in living organisms is shown. Many more CDNs have been discovered as described later in the review and illustrated in figure 4.

CDNs consist of two nucleotide bases cyclized in a ring formed from phosphodiester bonds between the ribose sugars (figure 1). These molecules control many aspects of bacterial physiology including biofilm formation, motility, virulence, stress responses and cellular development [1,2]. In this capacity, CDNs function as information carrier molecules, transducing the sensing of environmental conditions to the appropriate regulation of adaptive phenotypes [3]. In both bacteria and eukaryotes, some CDNs also function as danger signals to mediate cellular defences against biological conflict be it phage infection in bacteria or viral infection or cancer in eukaryotes. In bacterial cells, the synthesis of CDNs is triggered by phage infection, initiating various phage defence mechanisms to kill the infected cell or impede phage production [4–6]. Similarly, in a eukaryotic cell, CDN production is induced upon viral infection, triggering a Type I interferon (IFN) response via the receptor STING (Stimulator of IFN Genes) [7]. CDN production can also be activated when nuclear DNA leaks into the cytoplasm in stressed or cancerous cells [7]. This activation of STING by CDNs is being harnessed as a novel cancer immunotherapy as discussed in this review. There are several excellent reviews that highlight the clinical potential of novel CDN analogues that activate STING to enhance immune targeting of cancer [8–10]. As a microbiologist who has studied the physiological role of CDNs in bacteria, my goal for this review is to highlight the use and clinical potential of natural, biologically derived CDNs for cancer treatment by describing the discovery of CDNs, their biological functions and studies that have used natural CDNs as therapeutics to treat cancer in pre-clinical models. I will also highlight the diversity of CDNs and even cyclic tri-nucleotides (CTNs), and discuss the advantages and disadvantages of using these naturally occurring signalling molecules to activate STING as a new class of cancer immunotherapy.

## 2. In the beginning: the discovery CDNs in bacteria

The first CDN, 3′-5′, 3′-5′ cyclic di-GMP (c-di-GMP) (figure 1), was discovered by the laboratory of Moshee Benziman in 1987 as an allosteric activator of cellulose synthesis in the bacterium *Acetobacter xylinum* (currently named *Komagataeibacter xylinus*) [11–13]. Benziman's laboratory realized that *in vitro* cellulose synthesis was potently stimulated by GTP; however, GTP itself was not the molecular activator of the cellulose synthase complex [11]. Rather, through a series of exquisite biochemical studies, they discovered that an enzyme known as a diguanylate cyclase (DGC) combined two GTPs to form the dimeric c-di-GMP, which then directly bound and activated the cellulose synthesis enzyme [12].

Over the next decade, Benziman's laboratory laid the foundation for the CDN field, discovering other examples of DGCs and phosphodiesterase enzymes (PDEs) that degrade c-di-GMP [14,15]; however, it was not until the 2000s with the advent of whole-genome sequencing and a growing interest in the molecular mechanisms that control bacterial biofilm formation that the widespread nature of c-di-GMP was fully appreciated. Seminal studies in bacteria like *Vibrio cholerae* [16]*, Pseudomonas aeruginosa* [17] and *Caulobacter crescentus* [18] supported the general model that high intracellular concentrations of c-di-GMP promote a sessile, biofilm state while low levels promote a motile, planktonic existence [19]. Moreover, as more genome sequences became available, it was clear that c-di-GMP signalling systems are widespread in bacteria and predicted to be present in approximately 85% of all bacterial species [20]. Since this time, many novel functions and regulatory mechanisms controlled by c-di-GMP have been discovered, and I refer the readers to excellent recent reviews on this topic [21,22].

C-di-GMP reigned supreme as the only known CDN until the discovery in 2008 of 3′-5′, 3′-5′ cyclic di-AMP (c-di-AMP) in the bacterium *Bacillus subtilis* [23] (figure 1). C-di-AMP was discovered upon elucidation of the structure of DisA, a protein in *B. subtilis* that synthesizes c-di-AMP to halt sporulation upon sensing DNA damage [23]. C-di-AMP, although not as widely conserved in bacteria, is used by many Gram-positive species, and a smaller subset of Gram-negative bacteria, to primarily respond to osmotic stress by regulating ion and osmolyte transport [24,25]. Importantly, the invasive bacterial pathogen *Listeria monocytogenes* secreted c-di-AMP into the eukaryotic cell cytoplasm, a phenotype that was key in the discovery of the eukaryotic CDN receptor STING as discussed below [26,27].

The family of CDNs welcomed a new member in 2012 with the discovery of the hybrid 3′-5′, 3′-5′ cyclic GMP-AMP (3′3′-cGAMP) in the bacterium *Vibrio cholerae* [28] (figure 1)*.* 3′3′-cGAMP is synthesized by the enzyme DncV encoded on the unique VSP-1 genomic island found in the current 7th pandemic *V. cholerae* isolates [28]. Production of 3′3′-cGAMP alters bacterial motility, membrane biogenesis and virulence, although the molecular receptors of 3′3′-cGAMP were unknown [28]. The first protein receptor for 3′3′-cGAMP was described as the phospholipase, CapV, which directly binds to and is activated by 3′3′-cGAMP and is encoded adjacent to *dncV* [29]. Such activation is an altruistic suicide mechanism whereby bacteriophage infection induces DncV synthesis of 3′3′-cGAMP, activation of CapV and subsequent killing of the infected cell thereby preventing further phage replication to protect the neighbouring population [4].

## 3. Eukaryotic cells sense CDNs to induce a Type I IFN response

Before the discovery of c-di-AMP or 3′3′-cGAMP, hints emerged that CDNs uniquely impact eukaryotic cells. The first such observation was that c-di-GMP specifically killed H508 human colon cancer cells in culture but did not exhibit toxicity towards normal rat kidney and human neuroblastoma cells [30]. This study was the first to suggest that eukaryotic cells specifically sensed and responded to CDNs. Further analysis determined that c-di-GMP induced an inflammatory response in eukaryotic cells characterized by IL-12, IFN-gamma, and other cytokines and cell surface markers, and c-di-GMP also enhanced dendritic cell (DC) stimulation of T cells [31,32]. Such studies catalysed research to use c-di-GMP as a vaccine adjuvant, and it increased protective immune responses in several vaccine models including induction of mucosal immunity and protection against pneumococcal infection [33].

McWhirter *et al*. [34] found that the response of eukaryotic cells to c-di-GMP was analogous to their response to cytoplasmic double-stranded DNA. Furthermore, their results suggested the receptor for c-di-GMP was cytoplasmic as a much greater response to the molecule was observed when it was transfected in liposomes rather than added extracellularly as a free molecule. This seminal paper further demonstrated that the kinase TBK1 and transcription factor IRF-3 were central regulators in the response to c-di-GMP and showed that different cell types elicited heterogeneous responses to c-di-GMP [34].

**Figure 2.**
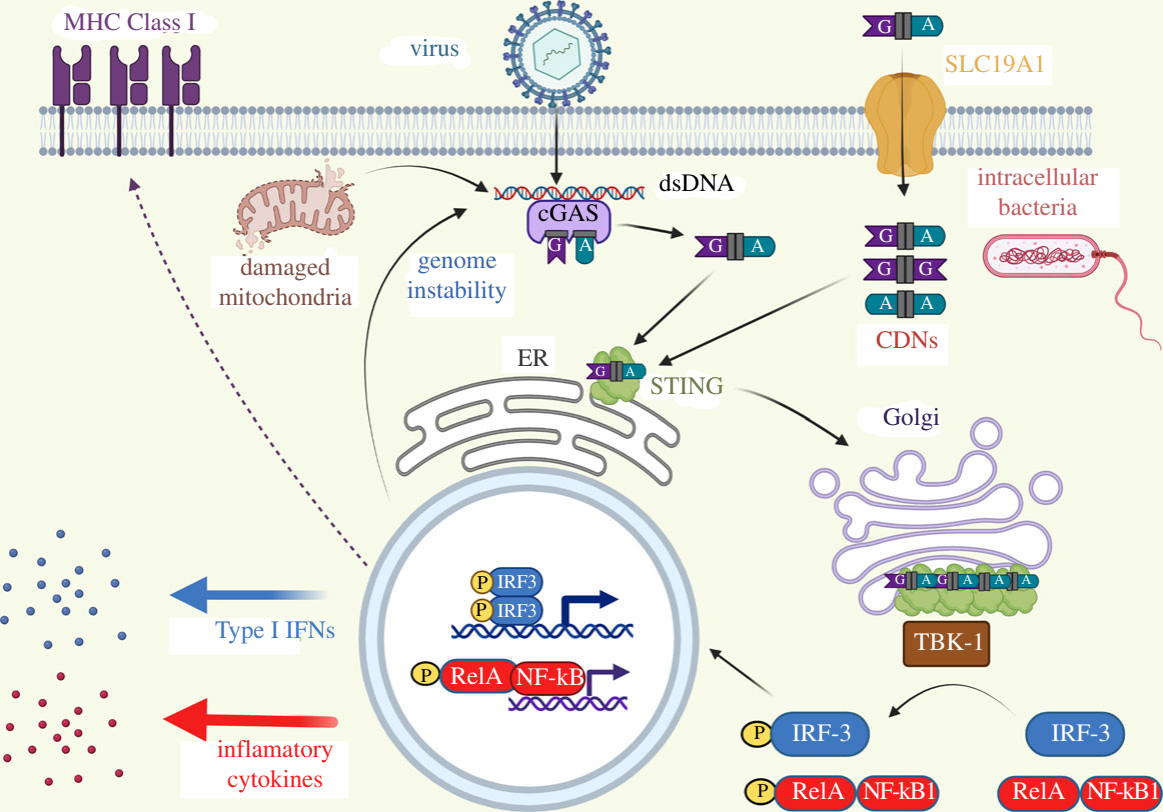
STING senses CDNs that are (i) synthesized by cGAS in response to cytoplasmic dsDNA from an infecting virus, damaged mitochondria, or genome instability, (ii) transported into the cell via the receptor SLC19A1 or (iii) secreted from invasive bacteria. Upon binding to CDNs, STING translocates to the Golgi and activates the kinase, TBK-1, ultimately inducing IRF-3 and canonical NF-κB regulated genes to induce MHC Class I expression on the cell surface and secretion of Type I IFNs and other inflammatory cytokines.

Confirming the predictions by McWhirter *et al*., two key studies demonstrated that STING is the CDN receptor responsible for the Type I IFN response in eukaryotic cells (figure 2). Building upon their previous findings, the Vance laboratory showed that expression of STING in HEK293 T cells, which do not normally respond to c-di-GMP, reconstituted induction of Type I IFNs in response to c-di-GMP addition [35]. Furthermore, purified STING directly bound to c-di-GMP. A study from the Portnoy laboratory supported these conclusions as STING deficient mice no longer induced a Type I IFN response to c-di-AMP secreted by intracellular *L. monocytogenes* [26,27]. Both c-di-GMP and c-di-AMP are predominantly synthesized in bacteria, with a few exceptions [36], suggesting that STING was capable of sensing CDNs synthesized from exogenous sources. Given the widespread nature of CDNs in bacteria, such recognition is reminiscent of a eukaryotic pattern recognition receptor that senses widely conserved microbial molecular signals to induce an inflammatory response [37].

STING was also implicated in the cellular response to double-stranded DNA, and it was unclear whether this response was distinct to its sensing of CDNs. However, these pathways merged with the discovery that the eukaryotic enzyme cGAS directly binds to dsDNA in the cell cytoplasm to synthesize 2′-5′, 3′-5′ cyclic GMP-AMP (2′3′-cGAMP) [38–43] (figure 1). 2′3′-cGAMP then binds to and activates STING triggering a molecular response that is highly analogous to activation by 3′3′-c-di-GMP and 3′3′-c-di-AMP [44] (figure 2). Thus, STING could be activated by both endogenous and exogenous CDNs; however, the ability of these CDNs to activate STING is not equivalent as the binding of these ligands to STING exhibits different dissociation constants (K_d_s) of 4.59 nM for 2′3′-cGAMP, 1.04 µM for 3′3′-cGAMP, 2.26–2.58 µM for c-di-AMP and 1.21 µM for c-di-GMP [42,45].

Upon recognition of CDNs, STING migrates from the endoplasmic reticulum to the Golgi and tetramerizes, recruiting the kinase TBK-1 to phosphorylate the transcription factor IRF-3 [46–48] (figure 2). STING activation can also activate the transcription factor canonical NF-κB, which is a heterodimer of RelA (p65) and NF-κB1 (p50) [49]. Phosphorylated IRF-3 and NF-κB translocate into the nucleus to induce a Type I IFN response as well as induce expression of MHC class I on the cell surface [8,49].

## 4. STINGing cancer: a new class of immunotherapy

The premise of cancer immunotherapy is that activation of the immune system can drive CD8+ cytotoxic T-lymphocytes (CTL) to recognize tumour-associated antigens (TAAs) and kill cancerous cells, thereby slowing, or even reversing, tumour growth. However, for a tumour to develop from a newly formed cancerous cell, it must evolve mechanisms to evade the normal host immune response. One such mechanism is the expression of the surface proteins PD-L1 or PD-L2 and CD80/CD86 on the cancer cells that bind and activate the checkpoint proteins PD-1 and CTLA-4, respectively, on T cells [50,51]. This recognition halts target cell killing by CTLs and can even induce their apoptosis or differentiation into immunosuppressive T regulatory cells [50,51]. The development of checkpoint inhibitors, monoclonal antibodies that bind to and block the interactions of PD-1 and CTLA-4 with their cognate ligands, inhibits this interaction stimulating tumour clearing in a subset of patients [52]. However, a significant number of cancer patients do not respond to checkpoint inhibitors, presumably because they have tumours with immunologically cold TMEs where CTLs are not activated or trafficked [53,54].

Activation of STING by CDNs has significant potential to enhance current cancer immunotherapy treatments by inducing inflammation in these immunologically cold tumours to synergize with checkpoint inhibitors. STING activation in the tumour micro-environment (TME) has many anti-cancer benefits [9]. Activation of STING in the cancer cells themselves leads to increased MHC class I expression, increasing display of TAAs leading to enhanced recognition of cancer cells by CTLs [55]. STING expressed in antigen-presenting cells (APCs) like DCs or macrophages can be directly activated by CDNs or indirectly activated via the Type I IFN response generated in the tumours [56–58]. These APCs migrate to lymph nodes where they cross-present TAAs to CTLs to mount an anti-cancer response [59]. STING enhancement in other cell types within or surrounding the tumour, such as endothelial cells, has also been implicated in anti-tumour responses [60]. This collection of activities makes STING agonists ideal for enhancing inflammation in the TME [10].

2′3′-cGAMP is synthesized intracellularly by cGAS in response to double-stranded DNA [38–43], but there are several mechanisms for cell-to-cell signalling via 2′3′-cGAMP in tumours. DC engulfment of cancer cells with elevated concentrations of 2′3′-cGAMP can activate STING in the DC via release of this CDN into the cell cytoplasm upon cellular degradation [61]. As cancer cells often have unstable nuclei with excess genomic DNA leaked into the cytoplasm, thereby activating cGAS to produce 2′3′-cGAMP, this might be a natural route to enhance immune targeting of tumours [56,62]. 2′3′-cGAMP can also spread from cell to cell via gap junctions in which cytoplasmic contents are exchanged [63] or transfer from epithelial cells to macrophages via connexins [64]. Finally, the folate receptor SLC19A1 is an importer for CDNs, providing a mechanism for extracellular CDNs in the TME to be imported by other cells [65,66] (figure 2). Furthermore, ionizing radiation stimulated increased 2′3′-cGAMP secretion from tumour cells [67]. However, extracellular 2′3′-cGAMP has a short half-life as it is degraded by the extracellular phosphodiesterase ENPP1 [67,68]. Furthermore, the release of AMP upon ENPP1 cleavage of adenine containing CDNs can be further metabolized by the surface exposed ecto-5′-nucleotidase CD73 to adenosine (ADO), which has immunosuppressive effects through binding to P2 purigenic receptors [69,70]. Therefore, CDNs in the extracellular milieu of a tumour are rapidly degraded, potentially into immunosuppressive signals, and inhibitors of ENPP1 are being developed to enhance STING activation [67].

The importance of STING for directing the immune system to recognize and target cancer is evident by studying the evolution of clinical tumours. Several studies observed that many cancers have evolved to reduce expression or otherwise inhibit STING activation. For example, functional STING activation was lost in 53.6% of malignant melanoma samples and 63.5% of metastatic samples [71]. Similar observations were made for human colorectal cancer samples, suggesting such evolutionary pressures are common to multiple cancer types [72]. Furthermore, STING activation may be central to the success of standard cancer treatments like radiation therapy or the DNA damaging agent cisplatin as these interventions are much less effective in STING deficient mice [73,74]. Thus, as further discussed below, one of the challenges of targeting STING in the clinic is overcoming these natural evolutionary processes that render STING signalling deficient in tumours.

## 5. Natural CDNs show promise in pre-clinical cancer models

With the high potential for STING activation to stimulate anti-cancer immune responses, multiple studies have demonstrated that treatment of tumours with CDNs inhibited or even reversed tumour growth in pre-clinical cancer models. In a seminal study, Fu *et al.* [75] showed that bacterial c-di-AMP and c-di-GMP could be formulated with lethally irradiated granulocyte–macrophage colony-stimulating factor (GM-CSF) vaccine cells derived from different tumour lines to generate what they referred to as STINGVAX treatments. STINGVAX administration demonstrated efficacy against multiple cancer models including B16, CT26, SCCFVII and Panc02. Importantly, c-di-AMP administration alone had no effect, likely due to the poor cellular uptake of free CDNs and their susceptibility to degradation by extracellular ENPP1 [75]. Likewise, intratumoral (IT) administration of 2′3′-cGAMP synergistically enhances radiation treatment of MC38 tumours in a STING-dependent manner, but administration of this CDN alone had no effect [74]. However, in some cases, the injection of free CDNs can enhance immune targeting of certain tumours. For example, IT administration of c-di-GMP into gliomas enhanced Type I IFN signalling, Ccl5 and Cxcl10 production while increasing CD4+ and CD8+ T cell infiltration [76]. Furthermore, intracranial injection of free c-di-GMP improved mouse survival [76]. IT injection of 2′3′-cGAMP also activated a STING-dependent anti-tumour immune response in melanoma and colon cancer models, and in some cases, such treatment synergized with checkpoint inhibitors [58,60]. In a similar study, IT injection of 2′3′-cGAMP increased vascularization of tumours in a STING-dependent manner, suggesting that increased access to the interior of the tumour might be one mechanism for synergy of CDNs with other immunotherapies [77].

The mixed outcomes observed with direct injection of free CDNs into tumours suggested this delivery method was not optimal. These results prompted the development of new methods of CDN delivery that could increase cellular entry while decreasing extracellular degradation. One such early attempt was the incorporation of c-di-GMP into pH-sensitive liposomes that demonstrated significant inhibition of E.G7-OVA and B16-F10 tumour growth [78,79]. 2′3′-cGAMP was also more effective when encapsulated in cationic liposomes with cholesterol polyethylene glycol surface coating, demonstrating STING induction *in vitro* in APCs and *in vivo* in a lung melanoma model [80]. This treatment also generated an immunological memory response which is optimal for treating metastatic cancer and increasing the chances of remission [80]. Encapsulation of 3′3′-cGAMP into hydrogenated (soy)L-α-phosphatidylcholine and 1,2-dioleoyl-3-trimethyl-ammonium-propane liposomes exhibited analogous activity in several *in vivo* cancer models [81]. Similarly, 2′3′-cGAMP loaded into these nanoparticles stimulated STING in neuroblastoma models triggering cell death and enhancing the response to a PD-1 checkpoint inhibitor [81]. An inhalable phosphatidylserine-coated liposome loaded with 2′3′-cGAMP activated APCs and inhibited tumour growth [82]. Combining these liposomes with radiation therapy stimulated systemic anti-cancer immunity [82]. 2′3′-cGAMP encapsulated in nanoparticles was demonstrated to have improved pharmacokinetic and pharmacodynamic (PK/PD) properties resulting in a 40-fold increase in stability, leading to enhanced T cell and PD-1 antibody infiltration into the TME [83].

In addition to liposomes, other nanoparticle delivery systems have been developed. c-di-GMP was encapsulated in cationic silica nanoparticles (CsiNPs) and administered to B16-F10 tumours [84]. The CsiNPs themselves can cause tumour cell death, leading to the release of TAAs, but their combination with c-di-GMP produced a synergistic effect with greater infiltration of APCs to the tumour leading to enhanced expansion of CD8+ T cells and enhanced tumour growth inhibition [84]. C-di-GMP was also loaded onto silica nanoparticles modified with poly(ethylene glycol) to enhance the immune response to 4T1 breast cancer cells, leading to suppressed tumour growth [85]. ‘nanoSTING-vax’ is a novel technology that mimics a cancer cell to concurrently deliver 2′3′-cGAMP and antigenic peptides, priming the immune system to recognize and target TAAs while enhancing the activity of checkpoint inhibitors [86].

Another major approach to overcome the inherent instability and poor cellular entry of CDNs is the development of chemical analogues that mimic CDNs but possess unique chemical features amenable to drug development. For example, synthesis of a bisphosphothioate analogue of 2'3'-cGAMP (2'3'-cG(s)A(s)MP) prevents degradation by ENPP1 up to 40-fold [68]. Three chemically synthesized STING agonists, ADU-S100 (clinical trial NCT02675439), MK-1454 (clinical trial NCT03010176) and E7766 (clinical trial NCT04144140) are currently being examined in clinical trials. As the focus of this review is the use of naturally occurring CDNs to treat cancer, I refer the reader to several excellent reviews that summarize the development of chemical CDNs analogues and other small molecule compounds to activate STING [9,10,87].

## 6. Five challenges to target STING with natural CDNs to prevent cancer

**Figure 3.**
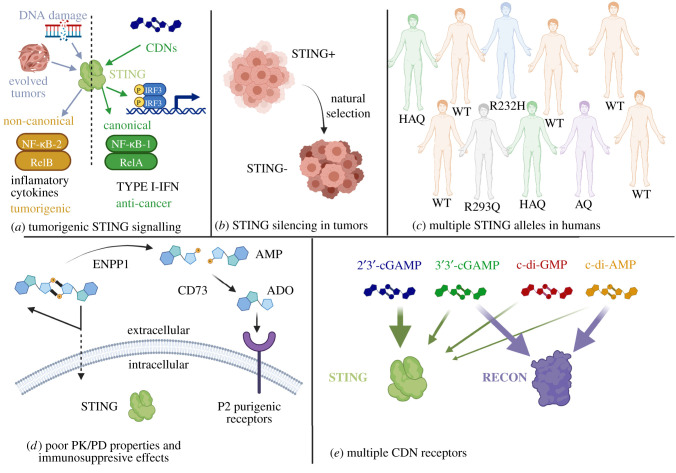
The five major challenges to targeting STING to treat cancer. (*a*) Some tumours and cell types have modified their STING signalling pathway such that activation of STING drives an inflamatory tumorigenic response through non-canonical NF-κB signalling. (*b*) A significant portion of tumours has silenced their STING pathway. (*c*) Humans have five major alleles of STING which interact differently with CDNs. (*d*) Free CDNs poorly cross cell membranes to access STING and are rapidly degraded by the extracellular PDE ENPP1, giving these molecules undesirable PK/PD properties for drug development. In addition, the degradation of adenine containing CDNs by ENPP1 increases the accumulation of immunosuppressive ADO. (*e*) Eukaryotic cells have multiple CDN receptors, and it is poorly understood how CDN interaction with these receptors in different cancer cells impacts anti-tumour immune responses.

These promising pre-clinical data have driven clinical trials of small molecules that activate STING [87]. However, the results of these clinical trials show poor efficacy, which is likely due to the complexities of STING signalling in tumours [9,88]. Listed below are five major challenges that must be addressed to realize the full potential of treating cancer by activating STING (figure 3).

### 6.1. STING signalling can drive pro-metastatic responses

It is now recognized that STING is a signalling hub that can be activated in multiple ways leading to different outcomes [8] (figure 3*a*). On one hand, Type I IFN-β signalling through IRF-3 and canonical NF-κB signalling (RelA/NF-κB1) is known to enhance MHC-I expression, immune targeting and tumour clearance [9]. However, in some cancers, activation of STING can induce non-canonical NF-κB signalling (RelB/NF-κB2), which can lead to inflammatory cytokine production that drives metastasis [89–91]. Activation of STING in a cGAS independent manner by DNA damage pathways also promotes NF-κB over IRF-3 signalling [92]. To add to this complexity, dosing of STING agonists is not trivial as, paradoxically, lower doses of the CDN mimic ADU-S100 injected into tumours produced higher levels of tumour-specific circulating CD8+ T-cells compared with higher doses [93]. Although high doses of ADU-S100 cleared tumours, this response was not dependent on CD8+ T-cells but was rather due to an ‘ablative’ mechanism that directly caused tumour cell death, and rechallenged animals were less effective at preventing tumour development. High doses of ADU-S100 also led to a systemic distribution of the agonist to other tumours. Alternatively, injection of lower concentrations of ADU-S100 promoted higher immunogenicity and abscopal effects when combined with a checkpoint inhibitor, and mice that survived the primary challenge were more resistant to rechallenge [93]. One potential reason for the negative correlation between dosing and a robust anti-tumour CD8+ T cell response is that hyper-activation of STING can drive apotosis of T and B cells, inhibiting the development of tumour-specific adaptive immune responses [94]. Finally, cGAS itself can be translocated into the nucleus to inhibit PARP1 repair of double-stranded DNA breaks, promoting cancer metastasis [95]. Therefore, simply developing small molecules that activate STING may be ineffective in some clinical tumours to drive anti-cancer immune responses versus pro-metastatic cancer pathways.

### 6.2. There is strong selection for tumours to silence STING signalling

One observation of STING deficient mice is that they have increased rates for the development of some tumour types such as colitis-associated cancer [96]. Furthermore, STING activation is a key component of effective radiotherapy and chemotherapy [73,74,97]. Therefore, there is strong selective pressure for tumours to evolve non-functional or altered STING signalling to overcome immune surveillance or chemotherapy (figure 3*b*). It is thus not surprising that many cancers do not have a functional STING signalling pathway, presumably because this pathway is an important natural defence system for the immune system to target and eradicate cancer [71,72]. Pre-clinical cancer models suggest that the activation of STING both in the cancer cells in addition to tumour-associated APCs and endothelium promotes maximum immune targeting of tumours [98–100]. Therefore, maximal clinical efficacy of STING agonists requires STING activation in the cancer cells themselves, which is challenging in the clinic given that many cancers have silenced their STING pathway.

### 6.3. Human populations have multiple STING alleles that respond differently to CDNs

Humans possess five major STING alleles with the wild-type ‘WT’ STING accounting for 57.9% of the population (figure 3*c*). The other major variants include the HAQ (R71H-G230A-R293Q, 20.4%), R232H (13.7%), AQ (G230A-R293Q, 5.2%) and R293Q (1.5%) [101]. These alleles exhibit different basal activities of STING with HAQ exhibiting the lowest activity and different responses to CDNs [101]. While WT STING can respond to 2′3′-cGAMP, 3′3′-cGAMP, c-di-GMP and c-di-AMP to varying extents, R232H and R293Q exhibited reduced induction by c-di-GMP, c-di-AMP and 3′3′-cGAMP while maintaining a robust response to 2′3′-cGAMP [101]. Knock-in mice of HAQ and R232H STING alleles have been generated and are useful tools to examine the *in vivo* activity of various STING agonists to these alleles *in vivo* [102]. Results from these mice generally support the *in vitro* findings that these STING alleles are less responsive to exogenous CDNs (3′3′-c-di-GMP, 3′3′-c-di-AMP and 3′3′-cGAMP). HAQ mice also exhibited a reduced response to endogenous 2′3′-cGAMP while the R232H mice robustly responded to this CDN [102]. Thus, the clinical application of any STING agonists must quantify their activity on these naturally occurring STING alleles.

### 6.4. Poor PK/PD properties of CDNs

Free CDNs have poor PK/PD properties due to their sensitivity to degradation by ENPP1 and inability to freely diffuse into target cells [67,68] (figure 3*d*). As mentioned, the degradation of adenine containing CDNs by ENPP1 can ultimately be converted to immunosuppressive ADO by CD73. Furthermore, given the potent nature of STING agonists, delivery is typically done via IT injection to limit systemic side effects, although there are CDN analogues being developed for potential systemic delivery [103,104]. Tumour retention time of such small molecules may be limited, leading to poorer efficacy, necessitating the need for alternate delivery mechanisms such as nanoparticles [10]. Indeed, IT injection of 500 μg of ADU-S100 was systemically distributed and detected in a distal, uninjected tumour [93].

### 6.5. STING is not the only eukaryotic CDN receptor

One often-overlooked facet of CDN signalling in eukaryotic cells is that STING is not the only eukaryotic CDN receptor (figure 3*e*). In 2017, McFarland *et al*. [105] described the discovery of a ‘reductase controlling NF-κB’, named RECON, isolated from mouse liver extracts that binds tightly to bacterially derived c-di-AMP and 3′3′-cGAMP, but not c-di-GMP or 2′3′-cGAMP. Ligand-free RECON inhibits NF-κB activation, but RECON itself is inhibited upon binding to CDNs, leading to activation of NF-κB and stimulation of an anti-bacterial inflammatory response. Thus, RECON is hypothesized to respond to bacterial CDNs to generate an anti-bacterial response distinctly different from the anti-viral/anti-cancer response generated by STING. RECON also negatively impacts STING signalling by sequestering free cytoplasmic CDNs [105]. Given that the activation of NF-κB can drive STING-dependent tumorigenic responses while inhibiting a Type I-IFN anti-cancer response, it is critical to understand how STING agonists not only impact STING signalling but also RECON signalling, which unfortunately is overlooked in virtually all studies on STING agonists. Importantly, the function of RECON as a CDN receptor has not yet been demonstrated in humans. In addition to RECON, the eukaryotic proteins DDX41 and ERAdP have also been described to sense CDNs and could be significant in modulating tumour responses to CDN therapeutics [106,107]. Finally, c-di-GMP induced cyclooxygenase 2 in a STING independent manner, suggesting that other eukaryotic CDN receptors remain to be discovered [108].

## 7. Advantages of using naturally occurring CDNs to activate STING

There has been extensive development of CDNs analogues to activate STING for cancer therapy. Such molecules have shown promising improvements in PK/PD properties, and other non-CDN analogues such as the amidobenzimidazole AZBI are even being developed for systemic delivery [103,104,109]. However, none of these chemical analogues have as yet demonstrated robust efficacy in clinical trials, suggesting a ‘silver bullet’ STING agonist has not yet been found (excellently reviewed in [10]). With the pre-clinical success of such analogues, is there a role for the clinical development of naturally occurring CDNs? Indeed, these biological signalling molecules have some inherent advantages for STING activation which warrant further exploration.

### 7.1. Expanding diversity of naturally occurring CDNs

**Figure 4.**
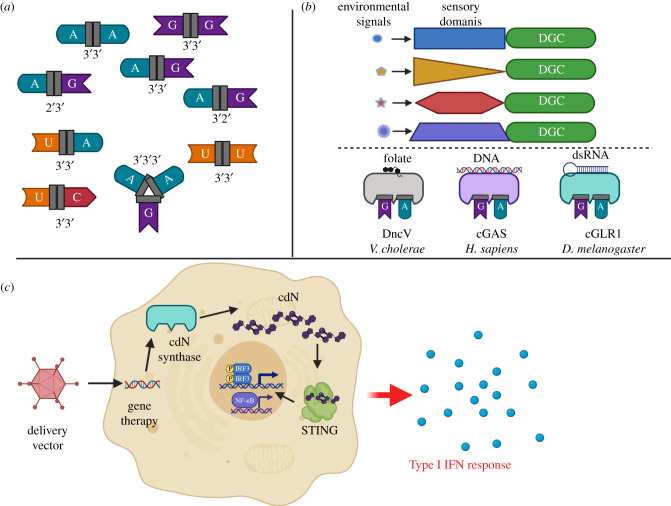
There are significant advantages for using naturally occurring CDNs to treat cancer. (*a*) Natural CDNs exhibit a wide diversity of nucleotide species and isomeric structures that could be exploited to activate the anti-cancer properties of STING in unique ways. (*b*) Since CDNs are synthesized by enzymes, they can be engineered to be active in response to specific environmental cues, as is seen for DGCs that synthesize c-di-GMP (top), or they can be regulated by their natural ligands, as shown for the CD-NTases (bottom). (*c*) Naturally occurring CDNs can be synthesized directly in target cells to activate STING using gene therapy approaches.

As summarized above, there are four major CDN signalling molecules in all living organisms: c-di-GMP, c-di-AMP and 3′3′-cGAMP in bacteria and 2′3′-cGAMP in metazoans (figure 1). However, one of the most exciting aspects in the field of CDN signalling is the recent discovery that bacteria and even eukaryotes synthesize a plethora of previously undiscovered CDNs and even CTNs, collectively referred to as cyclic oligo-nucleotides (CONs) [2] (figure 4*a*). Homologues of the cGAMP synthesis enzymes DncV from *V. cholerae* and cGAS from metazoans are widespread in bacteria and eukaryotes, and this family of enzymes has been named cGAS/DncV-like nucleotidyltransferase (CD-NTases) [110]. Interestingly, CD-NTases synthesize a higher diversity of CONs including the pyrimidine containing cyclic UMP-AMP, cyclic di-UMP, and even CTNs like cyclic AMP-AMP-GMP and cyclic tri-AMP. Moreover, diversity in cyclic ring linkages can also be found as 3′2′-cGAMP, which was shown to activate STING, was recently demonstrated to be synthesized by cGAS-like receptors cGLR1 and cGLR2 from the fruit fly *Drosophila melanogaster* [111,112]. Rather than binding and responding to dsDNA, cGLR1 is activated in response to dsRNA.

We now appreciate that natural CONs use a variety of different bases linked by both 2′-5′ and 3′-5′ phosphodiester bonds. This diversity has potential to activate STING in unique ways to maximize anti-tumour activities. Binding and activation of a subset CONs to STING has been directly assessed, and 2′3′-cGAMP is the most potent activator with cyclic UMP-AMP poorly activating STING [110]. The reverse is true for binding to RECON with 3′3′-cGAMP, c-di-AMP and to a less extent 3′3′-cyclic UMP-AMP binding to this receptor [110]. Cyclic AAG was not recognized by STING but did bind to RECON to activate NF-κB. There remain many other potential CONs that have been described or have the potential to be synthesized in living cells, and we are just beginning to understand the impact of these molecules on STING induction of Type I IFN and subsequent anti-cancer responses. One systematic analysis chemically synthesized every possible 3′3′-CDN and examined their ability to induce IFN-β production in a STING-dependent manner in RAW264.7-derived IFN regulatory factor reporter cells [113]. Their results suggest that c-di-GMP, c-di-AMP and 3′3′-cGAMP induce the strongest response, but it should be cautioned that these results only examined one concentration of CDN in one cell type and thus it remains to be determined if these results can be extrapolated to many cell types and TMEs [113]. Furthermore, as mentioned, there is not necessarily a direct correlation between STING activation and anti-cancer activity as higher concentrations of the STING-activating molecule ADU-S100 drove less immunogenic responses [93], and robust STING activation is lethal to some immune cells [94]. Therefore, the activity of these diverse CDN molecules in the TMEs, both in pre-clinical animal models and human tumours, must be further explored. Importantly, as it is well established that human tumours frequently mutate or alter the expression of their STING pathway, it would be informative to understand if the newer, more diverse set of CONs positively impact tumour immune surveillance through STING or other CON receptors like RECON.

### 7.2. Natural CONs are synthesized by enzymes amenable to gene therapy

Perhaps the most significant difference between naturally occurring CONs and chemical analogues is that the former can be generated in a biological cell by enzymes whereas the latter must be chemically synthesized. There are three families of enzymes that synthesize CONs. c-di-GMP, and more rarely 3′3′-cGAMP, is synthesized by DGCs, 3′3′-c-di-AMP is synthesized by diadenylate cyclases (DACs), while CD-NTases synthesize a wide array of CONs including 3′3′-cGAMP, 2′3′-cGAMP and 3′2′-cGAMP [2]. The primary substrates for all these enzymes are ribonucleotides, which are ubiquitous in all living cells. Furthermore, as these enzymes are active in the cell cytoplasm, they coexist in the same cellular compartment with STING itself. Delivery of DGCs, DACs or CD-NTases, or the genes that express them, into tumour-associated cells colocalizes CONs and STING in the cytoplasm. Such an approach overcomes two major roadblocks in the clinical development of STING agonists, poor cellular entry of CONs and susceptibility of extracellular CONs to ENPP1 degradation.

Another advantage of using enzymatic production of CONs in target cells to induce STING is that the synthase activity of these enzymes is allosterically regulated by cognate small molecules, allowing potential modulation of enzyme activity in a tumour-specific manner (figure 4*b*). For example, DGCs are modular proteins encoding a C-terminal DGC and N-terminal signalling domain [19,114,115]. As bacteria encode thousands of different DGCs, there is enormous genetic potential to identify DGCs that exhibit specific activity in certain cell types or tumour environments [116] (figure 4*b*, top). Likewise, CD-NTases are regulated by a variety of cellular factors. DncV from *V. cholerae* is repressed by cellular folates, while cGAS and the recently described cGLR1 are activated upon binding to dsDNA and dsRNA, respectively [40,111,112,117] (figure 4*b*, bottom). Further engineering using synthetic biology could link CON synthesis domains to different sensory domains to generate even more unique enzymes that exhibit beneficial activities in the proper environments or cell types. Such localized production of STING agonist is not possible with synthetic molecules, and this type of directed production of CONs specifically in the TME could minimize systemic toxic side effects. Finally, such a gene therapy approach also has the potential to limit the expression of CON synthase enzymes to tumours by genetic manipulation of their regulatory elements.

As the preponderance of efforts to activate STING has relied on injection of natural CDNs or other small molecule STING agonists, either free or incorporated into delivery systems like nanoparticles, there is little research exploring harnessing the power of CON synthases to activate STING (figure 4*c*). We have shown that the gene encoding two DGCs from the bacterial pathogen *V. cholerae* delivered into eukaryotic cells using an adenovirus type 5 delivery vector can synthesize high concentrations of intracellular c-di-GMP that robustly activates STING both *in vitro* in cell culture and *in vivo* in mouse models [118,119]. Such activation enhances both innate and adaptive immune responses. In a similar approach, the probiotic *Escherichia coli* Nissle auxotrophic mutant, designated strain SYNB1891, was engineered to deliver the CDN c-di-AMP into tumour-associated APCs [120]. Such delivery led to a significant inhibition of B16.F10 and A20 B cell lymphoma tumour growth, leading to a protective adaptive immune response in mice that had cleared the tumours [120]. Importantly, being a living cell, SYNB1891 represents a fabulous opportunity for genetic modification and engineering to further optimize this biological therapeutic for tumour inhibition.

Another gene therapy approach delivered a modified STINGS^162A/G230I/Q266I^ allele into cancer cells using adenovirus-associated vector-2 [121]. These cancer cells had reduced STING expression and thus poorly responded to STING agonists. The goal of this study was to develop a method that resensitized these cells to the STING agonist DMXAA [121]. DMXAA specifically activates mouse, but not human, STING. However, the human STINGS^162A/G230I/Q266I^ allele robustly responds to DMXAA, and cancer cells transfected with this gene now robustly responded to DMXAA. Such a gene therapy approach may allow for tumour-specific activation of STING signalling as non-transfected host cells will be DMXAA unresponsive [121].

Finally, an exciting study by Lam *et al*. [122] demonstrated that CDNs synthesized by the gut microbiota can enhance inflammation in the TME via Type I IFN signalling through STING activation. This anti-tumour response is primarily driven by increased populations of DCs, natural killer (NK) cells and anti-tumour macrophages. Diet also played a prominent role as a fibre-based diet promoted the growth of specific species of bacteria, including *Akkermansia muciniphila* which synthesized c-di-AMP, to activate mononuclear phagocytes through an unknown mechanism. Excitingly, the TME from patients that responded to checkpoint inhibitor treatment resembled the IFN-1/NK cell/DC profile of mice with CDN synthesizing microbiota while non-responders were more similar to germ-free mice, providing evidence that the results observed in mouse models were applicable to humans. Furthermore, faecal transplant of responders versus non-responders into mice showed a more inflamed TME and decreased tumour growth. This exciting study suggests that STING can be activated by natural CDNs in the gut by specifical microbial species, offering a new cornucopia of potential therapeutic innovations [122].

## 8. Summary

Activation of STING is a promising cancer immunotherapy that enhances immune targeting of tumours. Although clinical efforts in this area have focused on CDN analogues or other STING agonists, naturally occurring CONs have significant potential for clinical application, especially when incorporated into nanoparticles or other drug delivery platforms. One advantage of these naturally occurring molecules is their wide diversity, both in base composition and phosphodiester bonds, which may enhance the anti-tumour aspects of STING signalling. CON synthases can also be modified using synthetic biology approaches to limit activation to certain environments or cell types, and they can directly synthesize CDNs in target cells. Targeting STING to treat cancer is still a new field in its infancy. In addition to CDN analogues and other small molecule STING agonists, harnessing natural CON signals is one promising approach for this new cancer immunotherapy.

## Data accessibility

This article has no additional data.

## References

[1] Purificação AD, Azevedo NM, Araujo GG, Souza RF, Guzzo CR. 2020 The world of cyclic dinucleotides in bacterial behavior. Molecules **25**, 2462. (10.3390/molecules25102462) 32466317 PMC7288161

[2] Yoon SH, Waters CM. 2021 The ever-expanding world of bacterial cyclic oligonucleotide second messengers. Curr. Opin. Microbiol. **60**, 96-103. (10.1016/j.mib.2021.01.017) 33640793 PMC8026173

[3] Hengge R. 2020 Linking bacterial growth, survival, and multicellularity – small signaling molecules as triggers and drivers. Curr. Opin. Microbiol. **55**, 57-66. (10.1016/j.mib.2020.02.007) 32244175

[4] Cohen D, Melamed S, Millman A, Shulman G, Oppenheimer-Shaanan Y, Kacen A, Doron S, Amitai G, Sorek R. 2019 Cyclic GMP-AMP signalling protects bacteria against viral infection. Nature **574**, 691-695. (10.1038/s41586-019-1605-5) 31533127

[5] Ye Q, Lau RK, Mathews IT, Birkholz EA, Watrous JD, Azimi CS, Pogliano J, Jain M, Corbett KD. 2020 HORMA domain proteins and a trip13-like ATPase regulate bacterial cGAS-like enzymes to mediate bacteriophage immunity. Mol. Cell **77**, 709-722.e7. (10.1016/j.molcel.2019.12.009) 31932165 PMC7036143

[6] Lowey B et al. 2020 CBASS immunity uses CARF-related effectors to sense 3'-5'- and 2'-5'-linked cyclic oligonucleotide signals and protect bacteria from phage infection. Cell **182**, 38-49.e17. (10.1016/j.cell.2020.05.019) 32544385 PMC7728545

[7] Eaglesham JB, Kranzusch PJ. 2020 Conserved strategies for pathogen evasion of cGAS-STING immunity. Curr. Opin. Immunol. **66**, 27-34. (10.1016/j.coi.2020.04.002) 32339908 PMC7158794

[8] Vashi N, Bakhoum SF. 2021 The evolution of STING signaling and its involvement in cancer. Trends Biochem. Sci. **46**, 446-460. (10.1016/j.tibs.2020.12.010) 33461879 PMC8122033

[9] Jiang M et al. 2020 cGAS-STING, an important pathway in cancer immunotherapy. J. Hematol. Oncol. **13**, 81. (10.1186/s13045-020-00916-z) 32571374 PMC7310007

[10] Motedayen Aval L, Pease JE, Sharma R, Pinato DJ. 2020 Challenges and opportunities in the clinical development of STING agonists for cancer immunotherapy. J. Clin. Med. **9**, 3323. (10.3390/jcm9103323) 33081170 PMC7602874

[11] Ross P, Aloni Y, Weinhouse H, Michaeli D, Weinberger-Ohana P, Mayer R, Benziman M. 1986 Control of cellulose synthesis in *Acetobacter xylinum*. A unique guanyl oligonucleotide is the immediate activator of the cellulose synthase. Carbohydrate Res. **149**, 101-117. (10.1016/S0008-6215(00)90372-0)

[12] Ross P et al. 1987 Regulation of cellulose synthesis in acetobacter xylinum by cyclic diguanylic acid. Nature **325**, 279-281. (10.1038/325279a0) 18990795

[13] Romling U, Galperin MY. 2017 Discovery of the second messenger cyclic di-GMP. Methods Mol. Biol. **1657**, 1-8. (10.1007/978-1-4939-7240-1_1) 28889281 PMC5931213

[14] Ausmees N, Mayer R, Weinhouse H, Volman G, Amikam D, Benziman M, Lindberg M. 2001 Genetic data indicate that proteins containing the GGDEF domain possess diguanylate cyclase activity. FEMS Microbiol. Lett. **204**, 163-167. (10.1111/j.1574-6968.2001.tb10880.x) 11682196

[15] Chang AL, Tuckerman JR, Gonzalez G, Mayer R, Weinhouse H, Volman G, Amikam D, Benziman M, Gilles-Gonzalez M-A. 2001 Phosphodiesterase A1, a regulator of cellulose synthesis in *Acetobacter xylinum*, is a heme-based sensor. Biochemistry **40**, 3420-3426. (10.1021/bi0100236) 11297407

[16] Tischler AD, Camilli A. 2004 Cyclic diguanylate (c-di-GMP) regulates *Vibrio cholerae* biofilm formation. Mol. Microbiol. **53**, 857-869. (10.1111/j.1365-2958.2004.04155.x) 15255898 PMC2790424

[17] Simm R, Morr M, Kader A, Nimtz M, Römling U. 2004 GGDEF and EAL domains inversely regulate cyclic di-GMP levels and transition from sessility to motility. Mol. Microbiol. **53**, 1123-1134. (10.1111/j.1365-2958.2004.04206.x) 15306016

[18] Aldridge P, Paul R, Goymer P, Rainey P, Jenal U. 2003 Role of the GGDEF regulator PleD in polar development of *Caulobacter crescentus*. Mol. Microbiol. **47**, 1695-1708. (10.1046/j.1365-2958.2003.03401.x) 12622822

[19] Romling U, Galperin MY, Gomelsky M. 2013 Cyclic di-GMP: the first 25 years of a universal bacterial second messenger. Microbiol. Mol. Biol. Rev. **77**, 1-52. (10.1128/MMBR.00043-12) 23471616 PMC3591986

[20] Galperin MY. 2004 Bacterial signal transduction network in a genomic perspective. Environ. Microbiol. **6**, 552-567. (10.1111/j.1462-2920.2004.00633.x) 15142243 PMC1414778

[21] Hengge R. 2021 High-specificity local and global c-di-GMP signaling. Trends Microbiol. **29**, 993-1003. (10.1016/j.tim.2021.02.003) 33640237

[22] Valentini M, Filloux A. 2019 Multiple roles of c-di-GMP signaling in bacterial pathogenesis. Annu. Rev. Microbiol. **73**, 387-406. (10.1146/annurev-micro-020518-115555) 31500536

[23] Witte G, Hartung S, Büttner K, Hopfner KP. 2008 Structural biochemistry of a bacterial checkpoint protein reveals diadenylate cyclase activity regulated by DNA recombination intermediates. Mol. Cell **30**, 167-178. (10.1016/j.molcel.2008.02.020) 18439896

[24] Commichau FM, Dickmanns A, Gundlach J, Ficner R, Stülke J. 2015 A jack of all trades: the multiple roles of the unique essential second messenger cyclic di-AMP. Mol. Microbiol. **97**, 189-204. (10.1111/mmi.13026) 25869574

[25] Zarrella TM, Bai G. 2020 The many roles of the bacterial second messenger cyclic di-AMP in adapting to stress cues. J. Bacteriol. **203**, e00348-20. (10.1128/JB.00348-20) 32839175 PMC7723955

[26] Woodward JJ, Iavarone AT, Portnoy DA. 2010 c-di-AMP secreted by intracellular Listeria monocytogenes activates a host type I interferon response. Science **328**, 1703-1705. (10.1126/science.1189801) 20508090 PMC3156580

[27] Sauer JD et al. 2011 The N-ethyl-N-nitrosourea-induced Goldenticket mouse mutant reveals an essential function of Sting in the *in vivo* interferon response to *Listeria monocytogenes* and cyclic dinucleotides. Infect. Immun. **79**, 688-694. (10.1128/IAI.00999-10) 21098106 PMC3028833

[28] Davies BW, Bogard RW, Young TS, Mekalanos JJ. 2012 Coordinated regulation of accessory genetic elements produces cyclic di-nucleotides for *V. cholerae* virulence. Cell **149**, 358-370. (10.1016/j.cell.2012.01.053) 22500802 PMC3620040

[29] Severin GB et al. 2018 Direct activation of a phospholipase by cyclic GMP-AMP in El Tor *Vibrio cholerae*. Proc. Natl Acad. Sci. USA **115**, E6048-E6055. (10.1073/pnas.1801233115) 29891656 PMC6042076

[30] Karaolis DK, Cheng K, Lipsky M, Elnabawi A, Catalano J, Hyodo M, Hayakawa Y, Raufman JP. 2005 3′,5'-Cyclic diguanylic acid (c-di-GMP) inhibits basal and growth factor-stimulated human colon cancer cell proliferation. Biochem. Biophys. Res. Commun. **329**, 40-45. (10.1016/j.bbrc.2005.01.093) 15721270

[31] Karaolis DK et al. 2007 Bacterial c-di-GMP is an immunostimulatory molecule. J. Immunol. **178**, 2171-2181. (10.4049/jimmunol.178.4.2171) 17277122

[32] Karaolis DK, Newstead MW, Zeng X, Hyodo M, Hayakawa Y, Bhan U, Liang H, Standiford TJ. 2007 Cyclic di-GMP stimulates protective innate immunity in bacterial pneumonia. Infect. Immun. **75**, 4942-4950. (10.1128/IAI.01762-06) 17646358 PMC2044534

[33] Chen W, Kuolee R, Yan H. 2010 The potential of 3′,5'-cyclic diguanylic acid (c-di-GMP) as an effective vaccine adjuvant. Vaccine **28**, 3080-3085. (10.1016/j.vaccine.2010.02.081) 20197136

[34] McWhirter SM et al. 2009 A host type I interferon response is induced by cytosolic sensing of the bacterial second messenger cyclic-di-GMP. J. Exp. Med. **206**, 1899-1911. (10.1084/jem.20082874) 19652017 PMC2737161

[35] Burdette DL, Monroe KM, Sotelo-Troha K, Iwig JS, Eckert B, Hyodo M, Hayakawa Y, Vance RE. 2012 STING is a direct innate immune sensor of cyclic di-GMP. Nature **478**, 515-518. (10.1038/nature10429) PMC3203314 21947006

[36] Chen ZH, Schaap P. 2012 The prokaryote messenger c-di-GMP triggers stalk cell differentiation in *Dictyostelium*. Nature **488**, 680-683. (10.1038/nature11313) 22864416 PMC3939355

[37] Hangai S, Kimura Y, Taniguchi T, Yanai H. 2021 Signal-transducing innate receptors in tumor immunity. Cancer Sci. **112**, 2578-2591. (10.1111/cas.14848) 33570784 PMC8253268

[38] Li X et al. 2013 Cyclic GMP-AMP synthase is activated by double-stranded DNA-induced oligomerization. Immunity **39**, 1019-1031. (10.1016/j.immuni.2013.10.019) 24332030 PMC3886715

[39] Gao D, Wu J, Wu YT, Du F, Aroh C, Yan N, Sun L, Chen ZJ. 2013 Cyclic GMP-AMP synthase is an innate immune sensor of HIV and other retroviruses. Science **341**, 903-906. (10.1126/science.1240933) 23929945 PMC3860819

[40] Sun L, Wu J, Du F, Chen X, Chen ZJ. 2013 Cyclic GMP-AMP synthase is a cytosolic DNA sensor that activates the type I interferon pathway. Science **339**, 786-791. (10.1126/science.1232458) 23258413 PMC3863629

[41] Wu J, Sun L, Chen X, Du F, Shi H, Chen C, Chen ZJ. 2013 Cyclic GMP-AMP is an endogenous second messenger in innate immune signaling by cytosolic DNA. Science **339**, 826-830. (10.1126/science.1229963) 23258412 PMC3855410

[42] Zhang X, Shi H, Wu J, Zhang X, Sun L, Chen C, Chen Z. 2013 Cyclic GMP-AMP containing mixed phosphodiester linkages is an endogenous high-affinity ligand for STING. Mol. Cell **51**, 226-235. (10.1016/j.molcel.2013.05.022) 23747010 PMC3808999

[43] Kranzusch PJ, Lee ASY, Berger JM, Doudna JA. 2013 Structure of human cGAS reveals a conserved family of second-messenger enzymes in innate immunity. Cell Rep. **3**, 1362-1368. (10.1016/j.celrep.2013.05.008) 23707061 PMC3800681

[44] Gao P et al. 2013 Structure-function analysis of STING activation by c[G(2′,5′)pA(3′,5′)p] and targeting by antiviral DMXAA. Cell **154**, 748-762. (10.1016/j.cell.2013.07.023) 23910378 PMC4386733

[45] Pollock AJ, Zaver SA, Woodward JJ. 2020 A STING-based biosensor affords broad cyclic dinucleotide detection within single living eukaryotic cells. Nat. Commun. **11**, 3533. (10.1038/s41467-020-17228-y) 32669552 PMC7363834

[46] Zhang C, Shang G, Gui X, Zhang X, Bai X, Chen ZJ. 2019 Structural basis of STING binding with and phosphorylation by TBK1. Nature **567**, 394-398. (10.1038/s41586-019-1000-2) 30842653 PMC6862768

[47] Liu S et al. 2015 Phosphorylation of innate immune adaptor proteins MAVS, STING, and TRIF induces IRF3 activation. Science **347**, aaa2630. (10.1126/science.aaa2630) 25636800

[48] Zhao B et al. 2019 A conserved PLPLRT/SD motif of STING mediates the recruitment and activation of TBK1. Nature **569**, 718-722. (10.1038/s41586-019-1228-x) 31118511 PMC6596994

[49] Ishikawa H, Barber GN. 2008 STING is an endoplasmic reticulum adaptor that facilitates innate immune signalling. Nature **455**, 674-678. (10.1038/nature07317) 18724357 PMC2804933

[50] Sanaei MJ, Pourbagheri-Sigaroodi A, Kaveh V, Abolghasemi H, Ghaffari SH, Momeny M, Bashash D. 2021 Recent advances in immune checkpoint therapy in non-small cell lung cancer and opportunities for nanoparticle-based therapy. Eur. J. Pharmacol. **909**, 174404. (10.1016/j.ejphar.2021.174404) 34363829

[51] Ding L, Dong HY, Zhou TR, Wang YH, Yan T, Li JC, Wang ZY, Li J, Liang C. 2021 PD-1/PD-L1 inhibitors-based treatment for advanced renal cell carcinoma: mechanisms affecting efficacy and combination therapies. Cancer Med. **10**, 6384-6401. (10.1002/cam4.4190) 34382349 PMC8446416

[52] Robert C. 2020 A decade of immune-checkpoint inhibitors in cancer therapy. Nat. Commun. **11**, 3801. (10.1038/s41467-020-17670-y) 32732879 PMC7393098

[53] Majidpoor J, Mortezaee K. 2021 The efficacy of PD-1/PD-L1 blockade in cold cancers and future perspectives. Clin. Immunol. **226**, 108707. (10.1016/j.clim.2021.108707) 33662590

[54] He M, Yang T, Wang Y, Wang M, Chen X, Ding D, Zheng Y, Chen H. 2021 Immune checkpoint inhibitor-based strategies for synergistic cancer therapy. Adv. Healthc. Mater. **10**, e2002104. (10.1002/adhm.202002104) 33709564

[55] Falahat R, Perez-Villarroel P, Mailloux AW, Zhu G, Pilon-Thomas S, Barber GN, Mulé JJ. 2019 STING signaling in melanoma cells shapes antigenicity and can promote antitumor T-cell activity. Cancer Immunol. Res. **7**, 1837-1848. (10.1158/2326-6066.CIR-19-0229) 31462408 PMC6825582

[56] Schadt L et al. 2019 Cancer-cell-intrinsic cGAS expression mediates tumor immunogenicity. Cell Rep. **29**, 1236-1248.e7. (10.1016/j.celrep.2019.09.065) 31665636

[57] Torralba D et al. 2018 Priming of dendritic cells by DNA-containing extracellular vesicles from activated T cells through antigen-driven contacts. Nat. Commun. **9**, 2658. (10.1038/s41467-018-05077-9) 29985392 PMC6037695

[58] Li T et al. 2016 Antitumor activity of cGAMP via stimulation of cGAS-cGAMP-STING-IRF3 mediated innate immune response. Sci. Rep. **6**, 19049. (10.1038/srep19049) 26754564 PMC4709567

[59] Berraondo P, Minute L, Ajona D, Corrales L, Melero I, Pio R. 2016 Innate immune mediators in cancer: between defense and resistance. Immunol. Rev. **274**, 290-306. (10.1111/imr.12464) 27782320

[60] Demaria O et al. 2015 STING activation of tumor endothelial cells initiates spontaneous and therapeutic antitumor immunity. Proc. Natl Acad. Sci. USA **112**, 15 408-15 413. (10.1073/pnas.1512832112) PMC4687570 26607445

[61] Marcus A, Mao AJ, Lensink-Vasan M, Wang LA, Vance RE, Raulet DH. 2018 Tumor-derived cGAMP triggers a STING-mediated interferon response in non-tumor cells to activate the NK cell response. Immunity **49**, 754-763.e4. (10.1016/j.immuni.2018.09.016) 30332631 PMC6488306

[62] Turajlic S, Swanton C. 2016 Metastasis as an evolutionary process. Science **352**, 169-175. (10.1126/science.aaf2784) 27124450

[63] Ablasser A, Schmid-Burgk JL, Hemmerling I, Horvath GL, Schmidt T, Latz E, Hornung V. 2013 Cell intrinsic immunity spreads to bystander cells via the intercellular transfer of cGAMP. Nature **503**, 530-534. (10.1038/nature12640) 24077100 PMC4142317

[64] Pepin G et al. 2020 Connexin-dependent transfer of cGAMP to phagocytes modulates antiviral responses. mBio **11**, e03187-19. (10.1128/mBio.03187-19) 31992625 PMC6989113

[65] Luteijn RD et al. 2019 SLC19A1 transports immunoreactive cyclic dinucleotides. Nature **573**, 434-438. (10.1038/s41586-019-1553-0) 31511694 PMC6785039

[66] Ritchie C, Cordova AF, Hess GT, Bassik MC, Li L. 2019 SLC19A1 is an importer of the immunotransmitter cGAMP. Mol. Cell **75**, 372-381.e5. (10.1016/j.molcel.2019.05.006) 31126740 PMC6711396

[67] Carozza JA et al. 2020 Extracellular cGAMP is a cancer cell-produced immunotransmitter involved in radiation-induced anti-cancer immunity. Nat. Cancer **1**, 184-196. (10.1038/s43018-020-0028-4) 33768207 PMC7990037

[68] Li L, Yin Q, Kuss P, Maliga Z, Millán JL, Wu H, Mitchison TJ. 2014 Hydrolysis of 2'3'-cGAMP by ENPP1 and design of nonhydrolyzable analogs. Nat. Chem. Biol. **10**, 1043-1048. (10.1038/nchembio.1661) 25344812 PMC4232468

[69] Linden J, Koch-Nolte F, Dahl G. 2019 Purine release, metabolism, and signaling in the inflammatory response. Annu. Rev. Immunol. **37**, 325-347. (10.1146/annurev-immunol-051116-052406) 30676821

[70] Chang D, Whiteley AT, Bugda Gwilt K, Lencer WI, Mekalanos JJ, Thiagarajah JR. 2020 Extracellular cyclic dinucleotides induce polarized responses in barrier epithelial cells by adenosine signaling. Proc. Natl Acad. Sci. USA **117**, 27 502-27 508. (10.1073/pnas.2015919117) PMC7959571 33087577

[71] Xia T, Konno H, Barber GN. 2016 Recurrent loss of STING signaling in melanoma correlates with susceptibility to viral oncolysis. Cancer Res. **76**, 6747-6759. (10.1158/0008-5472.CAN-16-1404) 27680683

[72] Xia T, Konno H, Ahn J, Barber GN. 2016 Deregulation of STING signaling in colorectal carcinoma constrains DNA damage responses and correlates with tumorigenesis. Cell Rep. **14**, 282-297. (10.1016/j.celrep.2015.12.029) 26748708 PMC4845097

[73] Ahn J, Xia T, Konno H, Konno K, Ruiz P, Barber GN. 2014 Inflammation-driven carcinogenesis is mediated through STING. Nat. Commun. **5**, 5166. (10.1038/ncomms6166) 25300616 PMC4998973

[74] Deng L et al. 2014 STING-dependent cytosolic DNA sensing promotes radiation-induced type I interferon-dependent antitumor immunity in immunogenic tumors. Immunity **41**, 843-852. (10.1016/j.immuni.2014.10.019) 25517616 PMC5155593

[75] Fu J et al. 2015 STING agonist formulated cancer vaccines can cure established tumors resistant to PD-1 blockade. Sci. Transl. Med. **7**, 283ra52. 10.1126/scitranslmed.aaa4306 PMC4504692 25877890

[76] Ohkuri T, Ghosh A, Kosaka A, Zhu J, Ikeura M, David M, Watkins SC, Sarkar SN, Okada H. 2014 STING contributes to antiglioma immunity via triggering type I IFN signals in the tumor microenvironment. Cancer Immunol. Res. **2**, 1199-1208. (10.1158/2326-6066.CIR-14-0099) 25300859 PMC4258479

[77] Yang H et al. 2019 STING activation reprograms tumor vasculatures and synergizes with VEGFR2 blockade. J. Clin. Invest. **129**, 4350-4364. (10.1172/JCI125413) 31343989 PMC6763266

[78] Nakamura T, Miyabe H, Hyodo M, Sato Y, Hayakawa Y, Harashima H. 2015 Liposomes loaded with a STING pathway ligand, cyclic di-GMP, enhance cancer immunotherapy against metastatic melanoma. J. Control Release **216**, 149-157. (10.1016/j.jconrel.2015.08.026) 26282097

[79] Miyabe H, Hyodo M, Nakamura T, Sato Y, Hayakawa Y, Harashima H. 2014 A new adjuvant delivery system ‘cyclic di-GMP/YSK05 liposome’ for cancer immunotherapy. J. Control Release **184**, 20-27. (10.1016/j.jconrel.2014.04.004) 24727060

[80] Koshy ST, Cheung AS, Gu L, Graveline AR, Mooney DJ. 2017 Liposomal delivery enhances immune activation by STING agonists for cancer immunotherapy. Adv. Biosyst. **1**, 1600013. (10.1002/adbi.201600013) 30258983 PMC6152940

[81] Cheng N et al. 2018 A nanoparticle-incorporated STING activator enhances antitumor immunity in PD-L1-insensitive models of triple-negative breast cancer. JCI Insight **3**, e120638. (10.1172/jci.insight.120638) 30429378 PMC6302949

[82] Liu Y, Crowe WN, Wang L, Lu Y, Petty WJ, Habib AA, Zhao D. 2019 An inhalable nanoparticulate STING agonist synergizes with radiotherapy to confer long-term control of lung metastases. Nat. Commun. **10**, 5108. (10.1038/s41467-019-13094-5) 31704921 PMC6841721

[83] Wehbe M et al. 2021 Nanoparticle delivery improves the pharmacokinetic properties of cyclic dinucleotide STING agonists to open a therapeutic window for intravenous administration. J. Control Release **330**, 1118-1129. (10.1016/j.jconrel.2020.11.017) 33189789 PMC9008741

[84] An M, Yu C, Xi J, Reyes J, Mao G, Wei WZ, Liu H. 2018 Induction of necrotic cell death and activation of STING in the tumor microenvironment via cationic silica nanoparticles leading to enhanced antitumor immunity. Nanoscale **10**, 9311-9319. (10.1039/C8NR01376D) 29737353 PMC5969905

[85] Chen YP et al. 2020 STING activator c-di-GMP-loaded mesoporous silica nanoparticles enhance immunotherapy against breast cancer. ACS Appl. Mater. Interfaces **12**, 56 741-56 752. (10.1021/acsami.0c16728) 33305564

[86] Shae D et al. 2020 Co-delivery of peptide neoantigens and stimulator of interferon genes agonists enhances response to cancer vaccines. ACS Nano **14**, 9904-9916. (10.1021/acsnano.0c02765) 32701257 PMC7775800

[87] Le Naour J, Zitvogel L, Galluzzi L, Vacchelli E, Kroemer G. 2020 Trial watch: STING agonists in cancer therapy. Oncoimmunology **9**, 1777624. (10.1080/2162402X.2020.1777624) 32934881 PMC7466854

[88] Berry S et al. 2019 Correction to: 33rd Annual Meeting & Pre-Conference Programs of the Society for Immunotherapy of Cancer (SITC 2018). J. Immunother. Cancer **7**, 46. (10.1186/s40425-018-0422-y) 30760319 PMC6373015

[89] Bakhoum SF et al. 2018 Chromosomal instability drives metastasis through a cytosolic DNA response. Nature **553**, 467-472. (10.1038/nature25432) 29342134 PMC5785464

[90] Lemos H, Mohamed E, Huang L, Ou R, Pacholczyk G, Arbab AS, Munn D, Mellor AL. 2016 STING promotes the growth of tumors characterized by low antigenicity via IDO activation. Cancer Res. **76**, 2076-2081. (10.1158/0008-5472.CAN-15-1456) 26964621 PMC4873329

[91] Hou Y et al. 2018 Non-canonical NF-kappaB antagonizes STING sensor-mediated DNA sensing in radiotherapy. Immunity **49**, 490-503.e4. (10.1016/j.immuni.2018.07.008) 30170810 PMC6775781

[92] Dunphy G et al. 2018 Non-canonical activation of the DNA sensing adaptor STING by ATM and IFI16 mediates NF-κB signaling after nuclear DNA damage. Mol. Cell **71**, 745-760.e5. (10.1016/j.molcel.2018.07.034) 30193098 PMC6127031

[93] Sivick KE et al. 2018 Magnitude of therapeutic STING activation determines CD8^+^ T cell-mediated anti-tumor immunity. Cell Rep. **25**, 3074-3085.e5. (10.1016/j.celrep.2018.11.047) 30540940

[94] Gulen MF, Koch U, Haag SM, Schuler F, Apetoh L, Villunger A, Radtke F, Ablasser A. 2017 Signalling strength determines proapoptotic functions of STING. Nat. Commun. **8**, 427. (10.1038/s41467-017-00573-w) 28874664 PMC5585373

[95] Liu H et al. 2018 Nuclear cGAS suppresses DNA repair and promotes tumorigenesis. Nature **563**, 131-136. (10.1038/s41586-018-0629-6) 30356214

[96] Ahn J, Konno H, Barber GN. 2015 Diverse roles of STING-dependent signaling on the development of cancer. Oncogene **34**, 5302-5308. (10.1038/onc.2014.457) 25639870 PMC4998969

[97] Baird JR, Friedman D, Cottam B, Dubensky TW, Kanne DB, Bambina S, Bahjat K, Crittenden MR, Gough MJ. 2016 Radiotherapy combined with novel STING-targeting oligonucleotides results in regression of established tumors. Cancer Res. **76**, 50-61. (10.1158/0008-5472.CAN-14-3619) 26567136 PMC4703500

[98] Deutsch E, Chargari C, Galluzzi L, Kroemer G. 2019 Optimising efficacy and reducing toxicity of anticancer radioimmunotherapy. Lancet Oncol. **20**, e452-e463. (10.1016/S1470-2045(19)30171-8) 31364597

[99] Ko EC, Benjamin KT, Formenti SC. 2018 Generating antitumor immunity by targeted radiation therapy: role of dose and fractionation. Adv. Radiat. Oncol. **3**, 486-493. (10.1016/j.adro.2018.08.021) 30370347 PMC6200901

[100] Ngwa W, Irabor OC, Schoenfeld JD, Hesser J, Demaria S, Formenti SC. 2018 Using immunotherapy to boost the abscopal effect. Nat. Rev. Cancer **18**, 313-322. (10.1038/nrc.2018.6) 29449659 PMC5912991

[101] Yi G, Brendel VP, Shu C, Li P, Palanathan S, Cheng Kao C. 2013 Single nucleotide polymorphisms of human STING can affect innate immune response to cyclic dinucleotides. PLoS ONE **8**, e77846. (10.1371/journal.pone.0077846) 24204993 PMC3804601

[102] Walker MM, Kim S, Crisler WJ, Nguyen K, Lenz LL, Cambier JC, Getahun A. 2020 Selective loss of responsiveness to exogenous but not endogenous cyclic-dinucleotides in mice expressing STING-R231H. Front. Immunol. **11**, 238. (10.3389/fimmu.2020.00238) 32153571 PMC7049784

[103] Pan BS et al. 2020 An orally available non-nucleotide STING agonist with antitumor activity. Science **369**, eaba6098. (10.1126/science.aba6098) 32820094

[104] Chin EN et al. 2020 Antitumor activity of a systemic STING-activating non-nucleotide cGAMP mimetic. Science **369**, 993-999. (10.1126/science.abb4255) 32820126

[105] McFarland AP, Luo S, Ahmed-Qadri F, Zuck M, Thayer EF, Goo YA, Hybiske K, Tong L, Woodward JJ. 2017 Sensing of bacterial cyclic dinucleotides by the oxidoreductase RECON promotes NF-κB activation and shapes a proinflammatory antibacterial state. Immunity **46**, 433-445. (10.1016/j.immuni.2017.02.014) 28329705 PMC5404390

[106] Xia P et al. 2018 The ER membrane adaptor ERAdP senses the bacterial second messenger c-di-AMP and initiates anti-bacterial immunity. Nat. Immunol. **19**, 141-150. (10.1038/s41590-017-0014-x) 29292386

[107] Parvatiyar K et al. 2012 The helicase DDX41 recognizes the bacterial secondary messengers cyclic di-GMP and cyclic di-AMP to activate a type I interferon immune response. Nat. Immunol. **13**, 1155-1161. (10.1038/ni.2460) 23142775 PMC3501571

[108] Wang M, Chaudhuri R, Ong WW, Sintim HO. 2021 c-di-GMP induces COX-2 expression in macrophages in a STING-independent manner. ACS Chem. Biol. **16**, 1663-1670. (10.1021/acschembio.1c00342) 34478263

[109] Ramanjulu JM et al. 2018 Design of amidobenzimidazole STING receptor agonists with systemic activity. Nature **564**, 439-443. (10.1038/s41586-018-0705-y) 30405246

[110] Whiteley AT et al. 2019 Bacterial cGAS-like enzymes synthesize diverse nucleotide signals. Nature **567**, 194-199. (10.1038/s41586-019-0953-5) 30787435 PMC6544370

[111] Holleufer A et al. 2021 Two cGAS-like receptors induce antiviral immunity in *Drosophila*. Nature **597**, 114-118. (10.1038/s41586-021-03800-z) 34261128

[112] Slavik KM et al. 2021 cGAS-like receptors sense RNA and control 3'2'-cGAMP signalling in *Drosophila*. Nature **597**, 109-113. 34261127 10.1038/s41586-021-03743-5 PMC8410604

[113] Wang C, Sinn M, Stifel J, Heiler AC, Sommershof A, Hartig J. 2017 Synthesis of all possible canonical (3'-5'-linked) cyclic dinucleotides and evaluation of riboswitch interactions and immune-stimulatory effects. J. Am. Chem. Soc. **139**, 16 154-16 160. (10.1021/jacs.7b06141) 29056046

[114] Galperin MY. 2018 What bacteria want. Environ. Microbiol. **20**, 4221-4229. (10.1111/1462-2920.14398) 30187651 PMC7020242

[115] Hengge R, Gründling A, Jenal U, Ryan R, Yildiz F. 2016 Bacterial signal transduction by cyclic di-GMP and other nucleotide second messengers. J. Bacteriol. **198**, 15-26. (10.1128/JB.00331-15) 26055111 PMC4686208

[116] Chou SH, Galperin MY. 2016 Diversity of cyclic di-GMP-binding proteins and mechanisms. J. Bacteriol. **198**, 32-46. (10.1128/JB.00333-15) 26055114 PMC4686193

[117] Zhu D et al. 2014 Structural biochemistry of a *Vibrio cholerae* dinucleotide cyclase reveals cyclase activity regulation by folates. Mol. Cell **55**, 931-937. (10.1016/j.molcel.2014.08.001) 25201413

[118] Koestler BJ, Seregin SS, Rastall DPW, Aldhamen YA, Godbehere S, Amalfitano A, Waters CM. 2014 Stimulation of innate immunity by *in vivo* cyclic di-GMP synthesis using adenovirus. Clin. Vaccine Immunol. **21**, 1550-1559. (10.1128/CVI.00471-14) 25230938 PMC4248757

[119] Alyaqoub FS, Aldhamen YA, Koestler BJ, Bruger EL, Seregin SS, Pereira-Hicks C, Godbehere S, Waters CM, Amalfitano A. 2016 *In vivo* synthesis of cyclic-di-GMP using a recombinant adenovirus preferentially improves adaptive immune responses against extracellular antigens. J. Immunol. **196**, 1741-1752. (10.4049/jimmunol.1501272) 26792800 PMC5523134

[120] Leventhal DS et al. 2020 Immunotherapy with engineered bacteria by targeting the STING pathway for anti-tumor immunity. Nat. Commun. **11**, 2739. (10.1038/s41467-020-16602-0) 32483165 PMC7264239

[121] Liu W, Kim GB, Krump NA, Zhou Y, Riley JL, You J. 2020 Selective reactivation of STING signaling to target Merkel cell carcinoma. Proc. Natl Acad. Sci. USA **117**, 13 730-13 739. (10.1073/pnas.1919690117) PMC7306767 32482869

[122] Lam KC et al. 2021 Microbiota triggers STING-type I IFN-dependent monocyte reprogramming of the tumor microenvironment. Cell **184**, 5338-5356.e21. (10.1016/j.cell.2021.09.019) 34624222 PMC8650838

